# Prevalence and associated factors of depression among women with advanced pelvic organ prolapse in Northwest Ethiopia: cross-sectional study

**DOI:** 10.1186/s12905-024-03162-4

**Published:** 2024-05-30

**Authors:** Sileshi Ayele Abebe, Zelalem Mengistu Gashaw, Zelalem Ayichew, Dessie Abebaw Angaw, Endeshaw Asaye Kindie

**Affiliations:** 1 Department of Gynecology and Obstetrics, School of Medicine, College of Medicine and Health Sciences, University of Gondar, Gondar, Ethiopia; 2 Department of Public Health, School of Medicine, College of Medicine and Health Sciences, University of Gondar, Gondar, Ethiopia; 3 Department of Pathology, School of Medicine, College of Medicine and Health Sciences, University of Gondar, Gondar, Ethiopia

**Keywords:** Depression, Depressive symptoms, Pelvic organ prolapse, Urogenital prolapse, Vaginal vault prolapse

## Abstract

**Background:**

Depression is a symptom characterized by sadness, loss of interest or pleasure, feelings of guilt or low self-worth, disturbed sleep or appetite, feelings of tiredness and poor concentration. One of the most common mental illnesses in the world and a major contributor to morbidity and mortality is depression. The purpose of this study was to ascertain the prevalence of depression and the risk factors associated with it in women who had advanced pelvic organ prolapse.

**Methods:**

A facility-based cross-sectional study was conducted to determine depression among advanced pelvic organ prolapse women at Gondar University Comprehensive Specialized Hospital. All women who have advanced pelvic organ prolapse were consecutively included till it reached a total of 367 participants over four months. A structured questionnaire was used to obtain the sociodemographic characteristics, clinical characteristics and depression status of the participants. Depression measures were obtained by using the Patient Health Questionnaire tool, which is validated in the Ethiopian local language for chronic illnesses including pelvic organ prolapse using a cut point of five and above, which is considered to indicate depression. Women who screened positive were linked to a psychiatric clinic for further evaluation and treatment. Data was entered into a computer using Epi Info version 3.5.3 and then exported to STATA version 14 for analysis. Multivariable logistic regressions were fitted and odds ratios with 95% confidence intervals with a *P* value less than 0.05 were used to identify statistically significant factors.

**Results:**

The prevalence of depression was found to be 47.1% (95% CI: 43-52%). Being rural (AOR = 4.8; CI: 1.11–16.32), having a history of divorce because of pelvic organ prolapse (AOR = 5.5; CI: 1.85–16.32) and having a history of urinary symptoms (AOR = 3.1; CI: 1.12–8.59) were found to be independently associated with depression.

**Conclusions:**

The prevalence of depression among women with advanced pelvic organ prolapse in this study is high as compared to other studies. Depression screening strategies should be designed for the early identification and treatment of depression among women with advanced pelvic organ prolapse.

## Introduction

The global burden of depression is rising dramatically; as a result, approximately 322 million people are affected and living with it [1]. In 2008, the World Health Organization rated depression as the third leading cause of illness burden worldwide, with the disorder anticipated to rank first by 2030. The one-year prevalence of depression varies by country but is roughly 6% globally [1, 2].

Depression can result in considerable weight loss or gain, disrupted sleep, exhaustion, loss of appetite, feelings of hopelessness, low self-esteem and confidence, impaired thinking or concentration, and recurring thoughts of death. Worse, individuals may try suicide [1, 3, 4]. In addition, Women with depression have reduced physical, social, and occupational function [1, 5].

Pelvic organ prolapse (POP) is defined as the descent or displacement of a pelvic organ, which means the uterus, rectum, intestine, and bladder through the vagina from its normal anatomic position [6]. POP is a very common health problem in adult and elderly women and is a distressing condition [7].

The medical and social consequences of pelvic organ prolapse are distressing and can have a significant effect on mental health since depression has been associated with conditions that often accompany disability [8]. Having the pelvic organ outside the vagina hanging in between the thighs can morbidly affect a woman’s quality of life by limiting physical, social, psychological and sexual functions, and leading to depression [3, 6, 9].

Roughly one-third of postmenopausal women with symptomatic POP had depression. Depression was linked to lower urinary tract symptoms and altered bowel habits as a result of prolapse [10, 11]. Research indicates that POP and depressed symptoms are related, but there is insufficient data to draw a direct link between depression and advanced pelvic organ prolapse [7, 12, 13].

In Ethiopia, the burden of depression is not well-defined by using validated screening tools among patients with POP. Hence, this study will identify possible associated factors of depression among advanced POP patients using standard validated tools.

## Methods and materials

### Study design, study setting and period

An institution-based cross-sectional study was employed at the University of Gondar Comprehensive Specialized Hospital (UoGCSH) to assess the magnitude of depression and its associated factors among women with advanced pelvic organ prolapse from January 01 to April 30, 2023. Gondar City is located in the north-western part of Ethiopia and the western part of Amhara regional State. The city is located 741 km away from Addis Ababa, the capital city of Ethiopia, and 181 km away from Bahirdar, the capital city of the Amhara region. Gondar University Hospital, found in the central Gondar zone, is one of the largest tertiary-level referral and teaching hospitals in the Amhara Regional State and provides promotive, preventive, and curative services to over 5 million inhabitants in the catchment area [14, 15]. The urogynecology and fistula unit is one of the departments that provide services to 10–12 patients coming from Gondar town and its catchment area to the urogynecology and fistula unit per week. More than 430 patients are on the waiting list for pelvic organ prolapse surgery. The hospital also serves as a research centre and provides practical training for medicine and health science students.

### Population

#### Source population

All women who have advanced pelvic organ prolapse.

#### Study population

All women who have advanced pelvic organ prolapse during the study period.

### Inclusion and exclusion criteria

#### Inclusion criteria

All women diagnosed with advanced pelvic organ prolapse who visited Gondar University Hospital during the study period.

#### Exclusion criteria

Advanced pelvic organ prolapse women who were critically ill were exclude because it affected the outcome variable.

### Sample size determination and sampling procedure

The sample size was estimated using a single population proportion formula by assuming a 95% confidence level and 5% margin of error, and the prevalence of depression from a previous study performed in Ethiopia was 68% [3]. Considering a 10% nonresponse rate, the sample size was calculated using the formula n= (Z_a/2/)_^2^$\frac{\left[\text{P}(1-\text{P})\right]}{\text{d}2}$n= (1.96². (0.68(1-0.68)/ (0.05)2, *n* = 334, with 10%, no response rate; the sample size was 367. The sample size was also calculated for the second objective and the value was less than 367, so we used the final sample size based on the primary outcome. A consecutive sampling technique was applied to select all women who were clinically diagnosed with advanced pelvic organ prolapse by a urogynecologist at a urogynecology outpatient clinic until the needed sample size was reached.

### Study variables

#### Dependent variable

Depression.

#### Independent variables

Age, residency, number of children, number of stillbirths, educational status, marital status, chronic medical illness, duration of symptoms, urinary symptoms, bowel habit change (constipation), and type of urinary symptom.

### Operational definitions

#### Depression

We use PHQ-9 criteria which comprises nine items. Each item response is rated from “0” (not at all) to “3” (nearly every day), and the total score ranges from 0 to 27. A woman is said to have depression if she scores five and above out of the total score of 27 PHQ-9 tools and depression the total score ranges from 0 to 27. Depression is further classified based on severity, a score of 5 to 10 is mild, 10 to 20 is moderate, and greater than 20 represents severe depression [16].

#### Pelvic organ prolapse

Advanced pelvic organ prolapse was defined as urogynecologist-diagnosed stage III prolapse (the maximum descent of the prolapse halfway below the remnant of the hymnal ring) and stage IV complete prolapse of the pelvic organ (procidentia) based on POP-Q diagnosed tool [17–19].

### Data collection tools

The PHQ-9 was used to assess depression among pelvic organ prolapse patients. The PHQ-9 is a structured questionnaire that identifies depression using nine of the major depressive disorder symptoms from the DSM-V criteria. The PHQ-9 is commonly used for the screening of depression among chronic medical illnesses including POP [12, 20]. It is the validated tool in Ethiopia with a sensitivity of 88%, and specificity of 86% [16, 21, 22]. The tool was validated in Ethiopia and found to be reliable with Cronbach’s alpha of the scale was 0.98 and valid to assess depression and the structured questionnaire is available in English and the local language [23, 24]. The PHQ-9 comprises nine items, each item response is rated from “0” (not at all) to “3” (nearly every day), and the total score ranges from 0 to 27 [16]. A score of less than five represents no depression, a score of 5 to 10 is mild, 10 to 20 is moderate, and greater than 20 represents severe depression. For this study, we classified a woman as having depression if the PHQ-9 score is five and above and no depression if the score is less than five [22]. For the assessment of pelvic organ prolapse we use the most clinically used for the day to day evaluation and grading system. Which is a high interobserver agreement [19]. The remnant of the hymen is used as a fixed anatomic reference point. Zero indicates a normal anatomic position for a site, stage one indicates the maximum descent above the hymen, stage two indicates the level of a hymnal ring, stage three is the maximum descent halfway below the hymnal ring, whereas stage four represents maximum prolapse. The examination is performed with the patient straining so that maximum descent is attained. For this study, we use stage three and stage four as advanced pelvic organ prolapse [19, 25]. Structural questionnaires were prepared for sociodemographic and clinical characteristics of the participants based on previous literature and clinical practice.

### Data collection procedure and data quality control

Data were collected by two BSC female midwives after they had taken two days of data collection training. Standard questionnaire tools that included sociodemographic and clinical characteristics and PHQ-9 depression screening tools were prepared. This standard questionnaire was translated into the local language and back to English by two individuals who were fluent in both English and the Amharic language and who had experience in language translation. Before data collection began, a pretest was conducted on 5% of the sample size of a similar population which was not included in the final sample. The reason of the pretest is to reduce respondent burden, determine whether or not respondents are interpreting questions correctly, ensure that the order of questions is not influencing the way a respondent answers, and identify problems with the data collection instrument and questionnaire. Based on the results for the pretest, corrections and modifications were made by applying the questionnaire before applying it to the study area. Strict supervision was taken by the principal investigator. To ensure the quality of the data, the completed sheet was checked for completeness and consistency by the supervisor and the principal investigator. Additional rechecks and crosschecks of data entry were performed. Meanwhile, any doubts in the filled sheet are clarified for the data collector.

### Data processing and analysis

Data were entered into Epi Info version 3.5.4 and analysed using STATA software version 14. For descriptive statistics the median is used and categorical variables association between observed and expected were initially assessed using chi-square tests to summarize the results. Each variable was evaluated independently in a bivariable analysis, and associations were determined using cross-tabulation and COR at 95% CI. All variables associated with depression at a *p*-value < 0.25 in the bivariable logistic regression analysis were entered into a multivariable binary logistic regression analysis to control for confounders. The model of fitness was checked by the Hosmer and Lemeshow test and a *p*-value less than 0.05 was considered statistically significant.

### Ethical clearance

Ethical clearance was obtained from the institutional review board of the College of Medicine and Health Sciences, University of Gondar, with reference number 4017/2023. Permission was obtained from the Department of Obstetrics and Gynecology and the School of Medicine. Oral informed consent was obtained from each woman participating in the study. Privacy was maintained in a private room during the time of the interview, and confidentiality was maintained by storing data securely by passcode data access limited only to the researcher was also maintained throughout the data collection. After data collection, a counselling service was given at the institute in the area for those in need, and those women who screened positive were linked to a psychiatric ward for further evaluation and treatment and appointed for pelvic organ prolapse surgery.

## Results

### Sociodemographic characteristics of women with advanced pelvic organ prolapse

A total of 367 participants were interviewed, yielding a 100% response rate. The age of the study participants ranged from 26 to 75 years. The median age was 50 years. Among the study participants, 84.5% were rural dwellers, and approximately 28.9% of the participants were divorced; out of these 66% of women, the reason for divorce was the presence of POP. More than one-third of the study participants (35.2%) were illiterate, 53.1% of participants had two to four children, and 15.7% of the participants’ mothers had stillbirths (Table 1).

**Table 1:** Sociodemographic characteristics of women with advanced pelvic organ prolapse

Variables	Categories	Frequency(*n*)	Percent (%)
Age(year)	< _35	46	12.53
	36–49	125	34.06
	> _50	196	53.41
Residency	Urban	57	15.53
	Rural	310	84.47
Marital status	Married	250	68.1
	Divorced	106	28.9
	Single/Widowed	11	3.0
Educational status	illiterate	129	35.15
	1–6 grade	127	34.60
	7–12 grade	74	20.16
	College and above	37	10.08
Divorced because of pelvic organ prolapse	Yes	70	66.0
	No	36	34.0
Numbers of children	< _2	32	8.72
	2–4	195	53.13
	> _5	140	38.15
Stillbirth	Yes	56	15.26
	No	311	84.74

### Clinical characteristics of women with advanced pelvic organ prolapse

The median of symptoms among participants was 2 years, which ranged from 1 to 10 years. Approximately 16.35% of participants had a medical illness, and three-fourths of the participants had stage III prolapse at the time of evaluation. The prevalence of urinary compliance among participants was 40%. Of these, the most common symptom identified was difficulty with urination (52.4%), and nearly one-third (31.3%) of participants complained of urinary incontinence. More than half of the participants (60.76%) had compliance with constipation (Table 2).

**Table 2:** Clinical characteristics of women with advanced pelvic organ prolapse

Variables	Categories	Frequency(*n*)	Percent (%)
Medical illness	Yes	60	16.35
	No	307	83.65
Duration of symptoms	< _2	211	57.49
	3–5	124	33.79
	> _6	32	8.72
Stage of POP	Stage III	279	76.02
	Stage IV	88	23.98
Urinary symptom	Yes	147	40.05
	No	220	59.95
Type of urinary symptoms	Difficulty of urination	77	52.4
	Symptom of UTI	24	16.3
	Incontinence	46	31.3
Constipation	Yes	223	60.76
	No	144	39.24

### Prevalence of depression among women with advanced pelvic organ prolapse

The prevalence of depression among women who have advanced pelvic organ prolapse was 47.1% (95% CI, 43-52%). Out of 367 women with advanced pelvic organ prolapse, 173 of women reported depression. Most of them reported moderate depression (43.9%), mild depression (23.7%) and (32.4%) of the participant reported severe depression.

### Factors associated with depression among women with advanced pelvic organ prolapse

All variables were initially assessed with bivariable logistic regression, and those whose *P* values were less than 0.2 were analyzed with multivariable logistic regression to adjust for possible confounding variables. Variables such as residency, divorce because of the presence of pelvic organ prolapse, age of participants, and duration of symptoms, urinary symptoms and stage of prolapse were adjusted. The prevalence of depression among pelvic organ prolapse patients was highly associated with residency, urinary symptoms and divorce because of the presence of pelvic organ prolapse. Depressive episodes were more prevalent among rural residents than among urban residents, with an adjusted odds ratio of AOR = 4.8 (95% CI, 1.11–16.32). Patients who divorced because of the presence of pelvic organ prolapse had many more reported depression than those who divorced for other reasons (AOR 5.5; (95% CI, 1.85–16.32). Depression was identified more often in women who had urinary symptoms than in those who did not have urinary symptoms (AOR, 3.1; 95% CI, and 1.12–8.59) (Table 3).

**Table 3:** Bivariable and multivariable binary logistic regression analysis of factors associated with depression

Variables	At risk of depression			COR(95% CI)	AOR(95%CI)	*P* Value
	Yes	No	Total			
**Age** < _35 36–49 > _50	28	18	46	1		
	51	74	125	1.69(0.88–3.25)	0.3(0.53–1.94)	0.21
	94	102	196	0.75(0.48–1.18)	0.29(0.53–1.68)	0.17
**Marital status** Married Divorced Widowed/single	109	141	250	1		
	57	49	106	1.51(0.95–2.38)		
	7	4	11	2.26(0.65–7.93)		
**Educational status** College & above Grade 7–12 Grade 1–6 Uneducated	21	16	37	1		
	48	26	74	2.45(1.16–5.16)		
	59	68	127	3.45(1.89–6.27)		
	45	84	129	1.62(0.98–2.68)		
**Residency** Urban Rural	12	45	57	1		
	161	149	310	4.1(2.06–7.96)	4.8(1.11–16.32)	0.03
**Divorced b/c of POP** Yes No	47	23	70	5.3(2.2-12.86)	5.5(1.85–16.32)	0.002
	10	26	36	1		
**Number of children** < 2 2–4 > _5	12	20	32	1		
	93	102	93	1.52(0.7–3.28)		
	68	72	68	1.57(0.72–3.46)		
**Stillbirt**h Yes No	25	31	56	0.89(0.5–1.57)		
	148	163	311	1		
**Medical illness** No Yes	141	166	307	1		
	32	28	60	1.35(0.77–2.34)		
**Constipation** Yes No	70	74	144	1.1(0.72–1.68)		
	103	120	223	1		
**Urinary symptom** Yes No	96	51	147	3.5(2.26–5.42)	3.1(1.12–8.59)	0.003
	77	143	220	1		
**Urinary symptom type** Difficulty of urination	50	27	77	1		
UTI symptom	19	5	24	2.1(0.69–6.11)		
Incontinence	27	19	46	0.77(0.36–1.63)		
**Duration of Symptoms** <2 3–5 > _6	88	123	211	1		
	66	58	124	1.6(1.02–2.49)	4.1(0.26–13.24	0.18
	19	13	32	2.04(0.96–4.35)	3.7(0.83–17.29)	0.85
**Stage** III IV	125	154	279	1		
	48	40	88	1.5(0.91–2.39)	1.3(0.36–4.35)	0.71

P- Value < 0.05 = statically significant

## Discussion

The prevalence of depression and potential risk factors were evaluated in this study. The findings showed that women with advanced pelvic organ prolapse had a high percentage of depression symptoms. It was discovered that 47.1% of women with advanced POP had depression.

In terms of prevalence, our results are less than those of research conducted in Ethiopia twelve years prior (67.7%) [3]. This may be because access to health care to manage these conditions has significantly increased over time, and the duration of symptoms at the time of diagnosis was much shorter in our study (2 years) than in a previous study (3.5 years) [3]. Furthermore, currently, there has been a significant change among the population in the awareness of POP, disclosing their condition, and access to health care and treatment [26], which contributed to a decrease in the incidence of depression in this study [20, 25].

On the other hand, our results are greater than those of studies done in the United States of America, 22% [27], China 22.4% [28], Netherlands 30.9% [29] and Canada 37% [12]. This discrepancy could be due to the difference in socioeconomic status and the living standard of the study participants in which low socioeconomic status is 1.5 times the high risk of developing depression as compared to high socioeconomic status [1]. In addition they included stage II pelvic organ prolapse and other pelvic floor dysfunction which is not specific to advanced pelvic organ prolapse, which has less effect of depression as compared to advanced stage pelvic organ prolapse [30].

The likelihood of experiencing depression was strikingly elevated in those living in rural areas. The findings indicate that women with advanced POP in rural areas are more likely than those in urban areas to experience depression. This may be due to the sedentary lifestyles of rural residents and the physical demands of their jobs, as well as the fact that POP limits physical activity and worsens symptoms over time, which can lead to stress and depression [31, 32]. This implies identifying, and early treatment will decrease the burden of depression in those who have risk factors for the development of POP [26]. Since there are few studies relating POP to depression and living in a rural area, it is challenging to compare our findings with those of other researchers.

Compared to women without urinary complaints, women with POP-related urine symptoms had a higher chance of developing depression [7]. Which is consistent with previous studies done in China [28, 32]. This is because women who experience urine symptoms and pelvic organ prolapse are more likely to feel ashamed of their disease and fear discrimination from others in their immediate social circle lead to depression [7, 26]. This is better explained by the fact that women who have divorced have ongoing psychological stress and worry, which plays a significant role in the occurrence of depression [33–35].

This research endeavors to ascertain potential element that could be connected to the occurrence of depression. Recognizing and managing patients with advanced pelvic organ prolapse will greatly decrease the burden of depression. The **strength** of our study was data collected from the representative sample using the standard validated tool which gives a great insight into the burden of depression among advanced pelvic organ prolapse patients. The **limitation** of the study is that it is difficult to know the real cause-and-effect relationship between depression and advanced pelvic organ prolapse.

## Conclusions

There is a high prevalence of depression among women with advanced pelvic organ prolapse. In particular, being rural residents, being divorced because of pelvic organ prolapse and having urinary symptoms were independently associated with depression. Hence, careful assessment and early treatment of advanced pelvic organ prolapse patients will mitigate depression.

**Recommendations** for gynecologists: much attention should be given to women with pelvic organ prolapse and early identification and treatment of pelvic organ prolapse patients to prevent the occurrence of depression. Psychiatrists should screen women with advanced pelvic organ prolapse for early identification and treatment of depression. Further research is recommended to assess the prevalence of depression and to determine the cause-and-effect relationship between pop and depression.

## Data Availability

The datasets used and/or analysed during the current study are available from the corresponding author upon reasonable request.

## Abbreviations

AOR: Adjusted odds ratio
CI: Confidence Interval
COR: Crude odd ratio
DSM-V: Diagnosis and Statistical Manual of Mental Disorders
OR: Odds Ratio
PFD: Pelvic Floor Dysfunction
PHQ-9: Patient Health Questionnaire
POP: Pelvic Organ Prolapse
QOL: Quality of Life
UOGSH: The University of Gondar Specialized Hospital
WHO: World Health Organization
